# The Complement System and Its Role in Eosinophilic Inflammation in Respiratory Diseases

**DOI:** 10.3390/biomedicines14061363

**Published:** 2026-06-17

**Authors:** Zsófia Zdrobe, Ilona Tornyi, Anna Teréz Sárközi, Ildikó Horváth

**Affiliations:** 1 Department of Pulmonology, Faculty of Medicine, University of Debrecen, 4032 Debrecen, Hungary; zdrobezsofi@gmail.com (Z.Z.); sarkozi.anna@med.unideb.hu (A.T.S.); horvath.ildiko@med.unideb.hu (I.H.); 2 National Koranyi Institute of Pulmonology, 1122 Budapest, Hungary

**Keywords:** eosinophil inflammation, type-2 immunity, COVID-19, asthma, alarmins, lung cancer, barrier disease

## Abstract

The complement system is a key link between innate and adaptive immunity, contributing to pathogen elimination, immune regulation, and tissue homeostasis. Its activation is not only crucial in infections, such as COVID-19, but also plays a major role in the pathomechanism of several non-infectious respiratory diseases, such as asthma, COPD, sarcoidosis and lung cancer. Complement components can modulate the quality of the adaptive immune responses, including through the regulation of T2 immunity and eosinophilic inflammation, thereby linking natural defense to complex immune processes. In recent years, it has become increasingly clear that dysregulated complement activity contributes to inflammation, thrombosis and tissue damage in a wide range of respiratory diseases. The study of the various components of this cascade system may therefore be promising from both a diagnostic and therapeutic point of view. Some of its components may serve as biomarkers for distinguishing between different phenotypes of certain lung diseases, while their targeted inhibition or modulation may open the way towards new treatment options. A better understanding of the complement system’s integrative and regulatory role not only allows for a deeper insight into immunological interactions but may also bring us closer to phenotype-oriented, immunology-based pulmonology, which may have real clinical benefits in the future.

## 1. Introduction

Respiratory diseases represent a major global health burden, encompassing a wide spectrum of infectious and non-infectious conditions, from acute viral infections to chronic inflammatory disorders and malignancies. Although these entities differ in their clinical presentation, many share common immunopathological mechanisms, including epithelial barrier dysfunction, dysregulated inflammation, and maladaptive immune responses. Increasing evidence suggests that the complement system is critically involved in orchestrating these processes, positioning it not only as an effector arm of innate immunity but also as a central regulator of immune homeostasis in the airways.

The immune system relies on the tightly coordinated interaction between innate and adaptive immunity [1]. Innate immunity provides rapid, non-specific defence through cellular components such as macrophages and neutrophils, soluble mediators, and the complement system [1,2], while adaptive immunity generates antigen-specific responses and immunological memory through B and T lymphocytes [2,3]. Traditionally, these two arms were considered functionally distinct. However, it has now become clear that they are deeply interconnected, and the complement system represents a major interface between them [2].

The complement system is an evolutionarily conserved protein network composed of circulating and cell-associated components that can be rapidly activated in response to pathogens or tissue injury [4]. Although complement factors were long thought to be produced almost exclusively by the liver, it is now evident that they are also generated locally at barrier surfaces [4]. In the lung, epithelial cells, macrophages, fibroblasts and mesenchymal stem cells can all contribute to complement production, and several intracellular complement components have been identified in airway epithelial cells [4]. This local complement system is strategically positioned to participate in first-line defence and directly shape immune responses at the respiratory mucosa [2,4].

Complement activation contributes to host defence through opsonisation, phagocytosis, chemotaxis, and membrane attack complex formation [2,5,6]. These effects are mediated largely by the activation fragments C3a and C5a, which act as potent inflammatory and immunomodulatory mediators [2,5]. Beyond these classical antimicrobial functions, complement also actively regulates adaptive immunity by influencing antigen-presenting cell function, cytokine production, and T-cell polarisation [2,7,8]. In addition, antibody-dependent activation of the classical pathway provides a direct interface between adaptive humoral immunity and innate effector mechanisms [1,2].

Complement activation fragments function not only as inflammatory mediators but also as modulators of adaptive immunity. Anaphylatoxins such as C3a and C5a serve as signaling molecules linking innate immune activation to downstream adaptive responses, shaping subsequent T-cell responses [1,2].

Of particular relevance in respiratory disease is the ability of complement activation products to promote type 2 immune responses and eosinophilic inflammation [2]. Although neutrophilic inflammation can also be present in several respiratory diseases, eosinophils represent a central effector cell population and define clinically important disease phenotypes [9].

C3a and C5a act on different cell types, including neutrophils and eosinophils, both of which express receptors for these molecules [10]; however, the functional consequences of complement activation in eosinophil biology are less well studied.

C3a and C5a drive the production of IL-4, IL-5, and IL-13, thereby supporting Th2 polarisation and eosinophil recruitment. Eosinophils themselves express functional C3a and C5a receptors, enabling them to respond directly to complement activation with degranulation, release of cytotoxic mediators and sustained tissue inflammation [2]. In the context of airway epithelial injury, epithelial-derived alarmins such as IL-25, IL-33 and TSLP further amplify this complement-driven type 2 immune milieu. Together, these interactions define a complement–T2–eosinophil axis that represents a key pathogenic mechanism in allergic and chronic inflammatory airway diseases [2,11].

Complement activation has long been recognised as an essential component of antimicrobial defence during respiratory infections [8]. However, accumulating evidence indicates that its dysregulation is equally important in non-infectious lung conditions, including asthma, chronic obstructive pulmonary disease, sarcoidosis and lung cancer [5]. Excessive or inappropriate complement activity can promote persistent inflammation, tissue injury and immune imbalance, thereby fostering both acute and chronic respiratory pathology [5].

In this review, we summarise current knowledge on the role of the complement system in infectious and non-infectious respiratory diseases, with a particular focus on its function as a bridge between innate and adaptive immunity. We highlight emerging data supporting the importance of the complement–T2–eosinophil axis in airway inflammation, and discuss the diagnostic and therapeutic implications of targeting complement-mediated pathways in respiratory medicine. We also emphasize the potential of complement-based biomarkers and therapeutic strategies in advancing precision medicine approaches in pulmonology.

## 2. The Role of the Complement System in Infectious Respiratory Diseases

**Figure 1:**
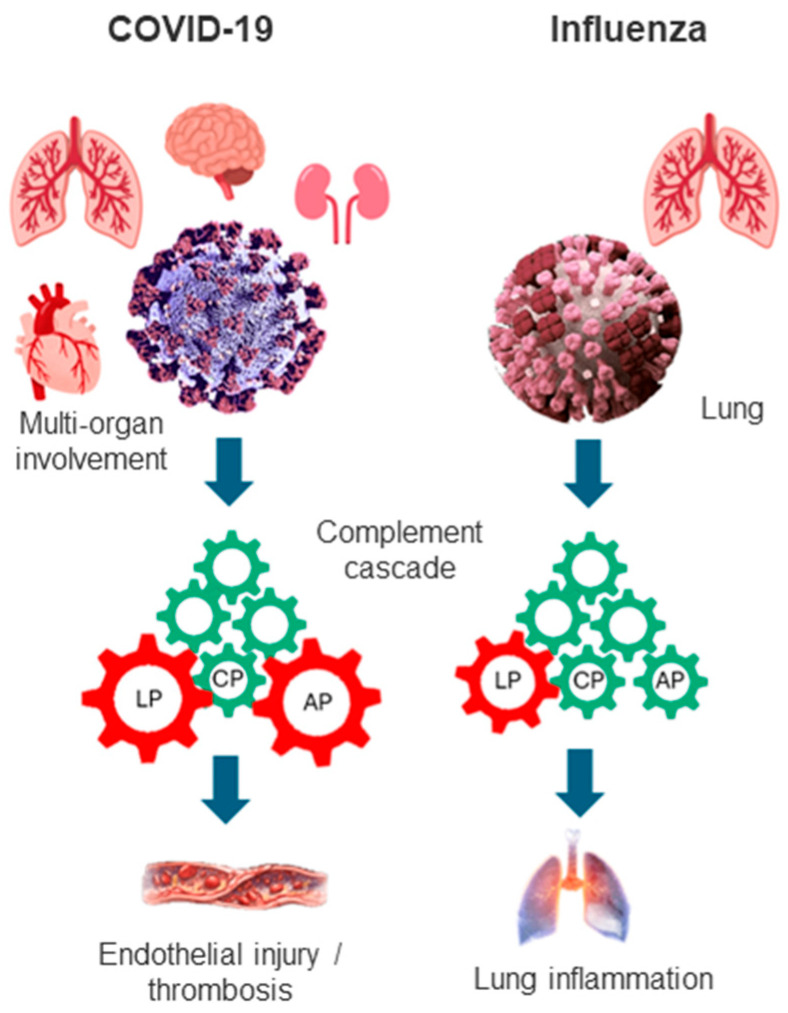
Comparison of COVID-19 and influenza infection from the perspective of the complement system.

The activation of the complement system is essentially one of the main complementary mechanisms of defense against pathogens [5]. This is evidenced by the fact that certain viruses have developed different mechanisms to prevent or exploit the functioning of the complement system to their advantage. Poxviruses, for example, produce a protein that is similar in structure and function to complement-regulating proteins, thereby silencing the cascade. A similar trend can be observed in flaviviruses, which avoid activation of the system by diverting the host’s complement-regulating proteins [8]. Previous research on the role of complement in influenza laid the foundation for later complement-focused studies related to COVID-19 infection (Figure 1).

Both SARS-CoV-2 and influenza viruses activate the complement system via the classical (CP), lectin (LP), and alternative (AP) pathways following interaction with airway epithelial cells. In COVID-19, the SARS-CoV-2 spike protein has been implicated in lectin pathway activation, and strong amplification via the alternative pathway predominates in severe disease, leading to enhanced C3 and C5 activation, endothelial injury and thrombosis. In influenza, complement activation is generally more balanced and supports antiviral clearance; however, in severe cases (e.g., H5N1) the lectin pathway may become dominant and contribute to tissue damage.

### 2.1. Influenza

Influenza is one of the most common acute respiratory infections, recurring seasonally and placing a significant burden on public health due to its high mortality rate.

One of the main tasks of our immune system is to provide protection against pathogens, so in the case of influenza infection, its primary role is to eliminate the virus. However, excessive activation of the system no longer helps with protection, but on the contrary, becomes harmful to the body. A significant proportion of patients with severe influenza-induced pneumonia require intensive care due to immune-mediated acute lung injury (ALI) and acute respiratory distress syndrome (ARDS) [12]. Based on research conducted in previous years, the complement system also plays a role in the development of these acute conditions through its overactivation [13].

The complement system is activated in all three pathways during influenza infection. The opsonisation of viruses by complement components, the promotion of virolysis by MAC, the enhancement of phagocytosis, and the modulation of the immune response through the production of chemotactic factors and anaphylatoxins support the elimination of the virus [13].

Excessive activation of the immune response, and thus ALI and ARDS, can be linked to increased complement system activity, particularly during H5N1 influenza infections. In one study, the products of complement activation and the expression of anaphylatoxin receptors were examined in the lungs of mice infected with the H5N1 virus. Their results showed that C3 deposition was increased and that complement activation occurred primarily via the lectin pathway. The use of a C3aR antagonist not only moderated the inflammatory response, reducing neutrophil infiltration and TNF-α levels, but also suppressed viral load. These results suggest that complement inhibition combined with antiviral therapy may be an effective strategy for treating ALI caused by H5N1 infection [13].

The complement protein C3, CD35/CD21 receptors (CR1/CR2) and IgM not only play a role in the immune response to influenza virus but also have a key role in the secondary immune response to influenza virus, i.e., the development of long-term memory against the pathogen [14]. Another study showed that human C1q, which is the recognition subunit of the classical pathway, significantly reduces the entry into cells and further replication of the H1N1 subtype of the influenza virus, while promoting the entry of the H3N2 subtype. C1q achieves this subtype-specific regulation without the involvement of other components of the complement system or antibodies. The main mechanism behind the complement-independent inhibition of H1N1 entry is likely to be the spatial barrier (steric inhibition) resulting from the large size of C1q. C1q interacts with haemagglutinin (HA) and neuraminidase (NA) proteins and is deposited on the surface of the influenza virus, thus masking the virus’s receptor-binding sites and preventing it from attaching to cells. This may represent a potential new therapeutic target in the early stages of infection, particularly in the defense against H1N1 [13,15].

### 2.2. COVID-19

The COVID-19 pandemic caused by the SARS-CoV-2 virus emerged at the end of 2019 and rapidly spread globally, posing unprecedented challenges to health systems and placing a significant burden on the daily functioning of societies [14]. COVID-19 is an acute viral respiratory disease with a highly heterogeneous clinical course, ranging from mild, self-limiting infection to severe pneumonia, acute respiratory distress syndrome (ARDS), multiorgan involvement and death. Early clinical observations indicated that disease severity could not be explained solely by viral load, suggesting that dysregulated host immune responses play a central role in driving severe outcomes [16,17]. Severe forms of COVID-19 had variable pathogenesis, but there is growing evidence that immune system overactivation is a major determinant of severe disease and high mortality [17].

**Figure 2:**
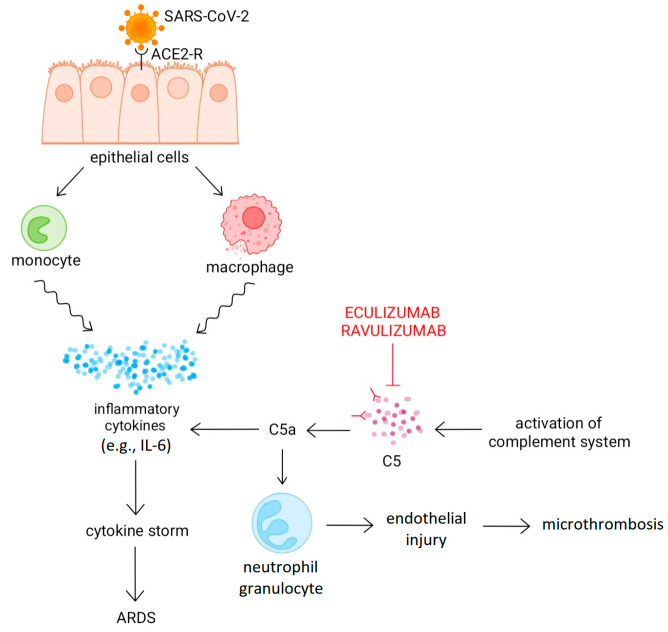
C5 inhibition as a therapeutic strategy in severe COVID-19. SARS-CoV-2 enters epithelial cells via the ACE2 receptor, triggering epithelial injury and innate immune activation. Complement activation leads to cleavage of C5 and generation of C5a. C5a promotes proinflammatory cytokine production by monocytes and macrophages (e.g., IL-6), contributing to cytokine storm and ARDS, and also drives neutrophil activation, resulting in endothelial injury and microthrombosis. Eculizumab and ravulizumab, monoclonal antibodies targeting C5, inhibit C5 cleavage and thereby attenuate complement-mediated inflammatory amplification in severe COVID-19.

Following the rapid spread of the pandemic, numerous studies focused on elucidating the pathogenesis of the infection. The results of these studies have made it clear that complement system activation, dysregulation of neutrophil granulocyte responses, endothelial damage and hypercoagulability collectively lead to severe clinical manifestations of COVID-19 [16]. Inflammatory mediators released during complement activation (C3a, C5a) are major drivers of neutrophil activation and sustained inflammation, while MAC causes endothelial and tissue damage, which directly contributes to thrombosis formation [16]. Proteomic analysis of plasma samples collected early in the pandemic showed that circulating sC5b-9 levels are significantly higher in COVID-19 patients requiring hospitalisation than in patients with influenza of similar severity or respiratory failure not associated with COVID-19. Furthermore, activation of the alternative pathway was observed to be particularly common in severe COVID-19 cases. Activation of this pathway was closely correlated with biomarkers of endothelial damage (angiopoietin-2) and hypercoagulability (thrombomodulin and von Willebrand factor). These results suggest that overactivation of the complement system may be one of the strongest indicators of COVID-19 infection [18]. In addition to the cytokine storm and ARDS playing a role in the development of respiratory failure and associated high mortality, endothelial dysfunction, platelet activation, and thrombosis of pulmonary capillaries also contribute to the devastating outcome. The central importance of complement system activation has been confirmed in thrombotic microangiopathy (TMA) associated with COVID-19, and complement-inhibiting therapeutic approaches have also been used, based on experience gained in the treatment of atypical haemolytic uraemic syndrome (aHUS) [19]. The concentrations of soluble factors in blood and bronchoalveolar lavage (BAL) fluid were examined in patients with SARS-CoV-2-infections of varying severity. The results showed a gradual increase in C5a levels as the disease progressed, and a significant increase in C5aR1 (CD88) receptor expression in peripheral blood. Histological studies also showed that the receptor was highly expressed in myeloid cells in the lungs, particularly in the vicinity of arteries and in thrombi. These observations suggest that the C5a–C5aR1 signaling pathway plays a key role in the development of ARDS [20]. Diurno et al. presented four severe COVID-19 cases in which supportive therapy was supplemented with eculizumab (Soliris^®^) treatment. Although the patients initially showed rapidly deteriorating respiratory function and bilateral milky infiltrates on chest CT scans, they all showed dramatic improvement within 48 h of receiving the first dose of eculizumab. Particularly noteworthy is the case of an elderly woman with multiple comorbidities who, despite severe lung damage, made a full recovery without mechanical ventilation [21]. Eculizumab is a humanised monoclonal antibody that inhibits the C5 component. Inhibition of C5 blocks only the final, endothelium-damaging stage of the complement system. The early complement components, which play a key role in modulating the immune response and recognising pathogens, remain functional. This allows for gentler yet effective immunomodulation [21]. Two European clinical trials are currently underway to investigate the use of eculizumab in COVID-19, and several studies are also underway to evaluate the efficacy of ravulizumab (Ultomiris^®^). Ravulizumab is also a recombinant C5 inhibitor antibody with a longer half-life than eculizumab [16] (Figure 2).

## 3. The Role of the Complement System in Non-Infectious Respiratory Diseases

### 3.1. Asthma

The incidence of allergic diseases is steadily increasing worldwide making asthma an increasingly significant public health challenge [2]. Asthma is one of the most prevalent chronic non-communicable respiratory diseases worldwide and remains a major contributor to morbidity despite substantial advances in diagnosis and therapy. As highlighted in recent comprehensive clinical reviews, asthma is no longer regarded as a uniform disease entity but rather as a heterogeneous condition encompassing diverse clinical phenotypes and underlying inflammatory endotypes [22,23]. This heterogeneity has become particularly relevant with the advent of targeted biological therapies, which have shifted asthma management towards a precision medicine approach based on disease mechanisms rather than symptoms alone [22,23]. Consequently, increasing emphasis has been placed on the identification of immunological pathways and clinically applicable biomarkers that can support patient stratification, guide treatment decisions, and improve long-term disease control [23]. Although no actual pathogens can usually be detected in the lung tissue of asthmatic individuals, pathological immunological processes are activated, leading to chronic inflammation and airway hyperresponsiveness. In the long term, these mechanisms result in the remodeling of the airways and lungs [22].

While in experimental asthma models, mice lacking Th2 cells fail to develop airway hyperresponsiveness (AHR), mucus hypersecretion, and eosinophilic responses [24], allergen-challenged C3a receptor-deficient mice and guinea pigs are similarly protected from bronchoconstriction and AHR [25]. Interestingly, no significant differences were observed in eosinophilic airway inflammation, Th2 cytokine production, or IgE levels between C3aR-deficient and wild-type animals. These findings provide compelling evidence that airway inflammation and AHR represent at least partially independent features of asthma [26,27,28]. Collectively, these findings suggest that complement activation, particularly via the C3a–C3aR axis, can modulate functional airway alterations independently of classical eosinophilic inflammation, highlighting its relevance as a potential therapeutic target in asthma [28].

**Figure 3:**
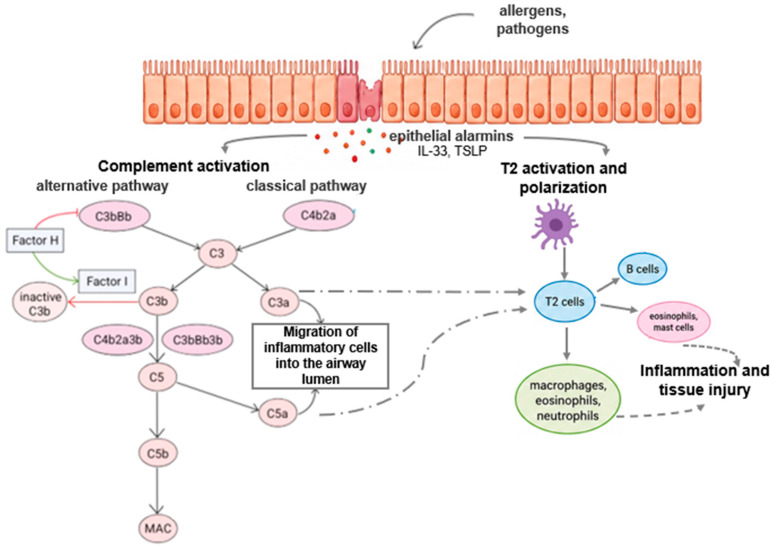
Complement and type 2 immunity interaction in asthma. Genetic predisposition and exposure to allergens or pathogens activate airway epithelial cells, initiating both complement activation and type 2 (T2) immune responses. The alternative pathway contributes to amplification of C3 activation. Cleavage of C3 and C5 generates the anaphylatoxins C3a and C5a, which promote migration of inflammatory cells into the airway lumen and contribute to epithelial injury through membrane attack complex (MAC) formation. Complement activity is regulated by factor H and factor I to limit excessive activation. Complement-derived anaphylatoxins and T2 cytokines together amplify airway inflammation, establishing a self-perpetuating inflammatory loop.

Although the adaptive immune system dominates in asthma through type 2 immune responses, and most research in this field focuses on acquired immunity, it is becoming increasingly clear that innate immune mechanisms also play a significant role in the pathomechanism [2,22]. Complement activation is linked to type 2 immune response-mediated signaling pathways, as levels of various complement factors, including C3 and H factor, correlate with eosinophil counts [29,30]. However, its role is not restricted to this immunological pathway, as it does not fit into any of the asthma groups defined based on the protein profiles or clinical subtypes [22] (Figure 3).

Several previous experimental studies have clearly confirmed the activation of the complement cascade in different models of asthma. It should be noted, however, that only a limited amount of data from human studies is available to date. Although the research methods vary, based on our own and others’ research results, it can be concluded that in samples of asthmatic patients, the levels of complement-regulating proteins (factor I and its cofactor, factor H), especially factor H, are higher than in healthy control groups [22,29].

Furthermore, in a population-based study involving more than 100,000 participants, Vedel-Krogh et al. demonstrated that higher C3 levels are associated with more severe forms of asthma and the frequency of hospital treatments [30]. This finding was complemented by an analysis that confirmed elevated C9 and I factor expression in the blood of a large number of severe asthma patients through proteomic testing [31].

In clinical practice, the identification of inflammatory endotypes relies on feasible and non-invasive biomarkers [23]. Fractional exhaled nitric oxide (FeNO) measurement has emerged as a validated tool for assessing airway inflammation, particularly in type 2–high asthma. The clinical feasibility and reproducibility of FeNO assessment using portable devices have been well established, reinforcing its role in asthma phenotyping [32].

However, the complement system is studied not because of a lack of established biomarkers, but because currently available markers predominantly reflect downstream inflammatory activity rather than the upstream regulatory processes that contribute to disease persistence. Based on the above, complement factors may serve as additional biomarkers for distinguishing between subgroups of asthma patients, ultimately supporting the development of more personalized therapeutic strategies [22]. Furthermore, in addition to the biological therapies already available, treatments that influence the complement system may offer new possibilities in asthma management [33].

### 3.2. Chronic Obstructive Pulmonary Disease (COPD)

Chronic obstructive pulmonary disease (COPD) is also characterized by persistent inflammation of varying intensity in the airways and a gradual deterioration in respiratory function. The inflammation flares up periodically, with these exacerbations usually triggered by respiratory tract infections [5].

It is well known that smoking plays a clear role in the development of COPD. A study using a mouse model investigated whether the C3 complement component modulates cigarette smoke-induced oxidative stress and apoptosis in the bronchial epithelium and whether it is involved in the pathogenesis of COPD. Elevated intracellular C3 levels were measured in samples from patients with end-stage COPD. Knockout of C3 exacerbated oxidative stress and apoptosis in 16HBE (human bronchial epithelial cell line) cells exposed to tobacco smoke in vitro. This correlation may indicate a protective effect of C3 in COPD [34].

Furthermore, it has been shown that serum and sputum levels of C3a, C4a and C5a are higher in stable (i.e., not exacerbated) COPD patients, suggesting that the complement system remains persistently active in COPD patients [35].

**Figure 4:**
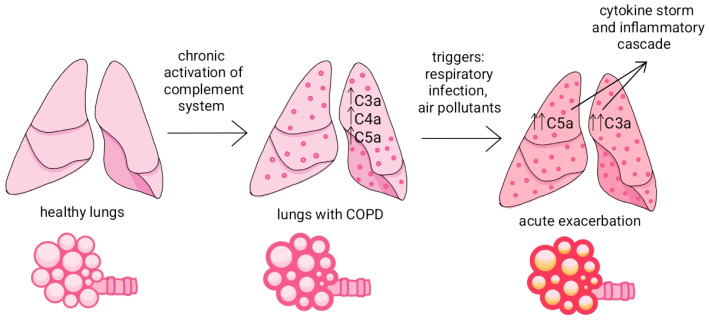
Elevated complement components in acute exacerbation of COPD. In healthy lungs, complement activity is tightly regulated. In stable COPD, chronic exposure to noxious stimuli leads to chronic activation of the complement system, resulting in elevated levels of complement activation products, including C3a, C4a and C5a, which contribute to ongoing airway inflammation and tissue injury. During acute exacerbations of COPD, triggered by respiratory infections or air pollutants, complement activation is further amplified. Increased generation of the anaphylatoxins C3a and C5a promotes excessive inflammatory responses, contributing to cytokine release, inflammatory cascade activation and worsening lung injury.

Another study pointed out that during exacerbations, sputum C3a and C5a levels are higher than at rest, and their increase correlates with the severity of the exacerbation [36], so the measurable levels of these complement components in sputum may be used as prognostic factors for exacerbation in the future (Figure 4).

In addition, Zhang et al. observed significantly lower C1q levels in serum samples from COPD patients than in non-COPD controls [37]. The decrease in C1q levels was clearly associated with decreased lung function (decreased Tiffeneau index (FEV1/FVC)) and worsening COPD [38].

In a recent case report, Kaneko et al. presented the case of a 70-year-old COPD patient with a long history of smoking who developed eosinophilic pneumonia in addition to hypocomplementaemic urticarial vasculitis syndrome (HUVS). HUVS is a rare, autoimmune vasculitis in which the components of the classical complement pathway (particularly C1q and C3) are low, and the condition is often associated with obstructive lung disease. Idiopathic chronic eosinophilic pneumonia (CEP), on the other hand, is a rare disease of unknown etiology, often associated with asthma, and is therefore thought to be caused by allergic mechanisms; its development is not usually associated with smoking. The low C3 and C1q levels observed in the presented case, as well as the simultaneous appearance of skin and lung symptoms, suggest that complement system dysregulation may play a role not only in vasculitis associated with immune complex formation, but also in the development of eosinophilic alveolar inflammation. The results suggest a possible link between HUVS, chronic eosinophilic pneumonia and long-term smoking [38].

In COPD, chronic epithelial injury and recurrent infections maintain this activity, promoting neutrophil recruitment, tissue damage, and increased exacerbation severity [5,35,36]. Experimental and clinical findings indicate that chronic complement activation is a hallmark of COPD and likely contributes to disease progression [35,36]. Accordingly, complement components and activation fragments have emerged as potential biomarkers of disease activity and prognosis [37]. However, despite accumulating evidence supporting a pathogenic role for complement dysregulation in COPD, complement-targeted therapeutic strategies have not yet entered routine clinical practice, underscoring the need for further translational and clinical studies, particularly in view of safety considerations related to host defense.

### 3.3. Sarcoidosis

Sarcoidosis is a rare granulomatous inflammatory disease of unknown etiology that primarily affects the chest organs, particularly the lung parenchyma and regional lymph nodes [39].

Sarcoidosis is thought to arise from the interaction of genetic susceptibility and environmental exposures, leading to a persistent and dysregulated immune response. Certain HLA associations, female sex, and ethnic background, together with infectious or environmental antigens, may trigger innate immune activation in the lower airways. Toll-like receptor signaling in alveolar macrophages, dendritic cells, and epithelial cells activates NF-κB–dependent inflammatory pathways and promotes a Th1-dominant adaptive immune response characterized by increased production of interferon-γ and tumor necrosis factor-α (TNF-α). These mediators drive granuloma formation and maintenance through sustained macrophage activation and recruitment [39].

With chronic disease progression, prolonged inflammation can lead to parenchymal damage and fibrotic remodeling, accompanied by a partial shift toward Th2-associated, profibrotic immune pathways [39]. Within this context, the complement system may become locally activated, both as a consequence of ongoing innate immune stimulation and through pattern-recognition molecules such as pentraxins, thereby contributing to the persistence of granulomatous inflammation [40].

Although the pathogenesis of the disease is still not fully understood, recent research has increasingly focused on the possible role of the complement system disease persistence [41,42]. Early human studies already indicated that local complement activation occurs in the lower airways. Analyses of bronchoalveolar lavage samples revealed no detectable functional activity of the classical complement pathway in healthy control subjects, whereas significant complement activity was observed in interstitial lung diseases, particularly in sarcoidosis. The magnitude of complement activity in sarcoidosis was markedly higher than that detected in idiopathic pulmonary fibrosis and decreased in response to corticosteroid therapy. These findings suggest that in sarcoidosis, complement is not merely passively present in the airways but is functionally active and may contribute to the immunopathogenesis of the disease [43].

Proteomic analyses have shown that complement-activating proteins, mainly factor B, C1q and C3, are significantly enriched in exosomes isolated from BAL samples of sarcoidosis patients, while the expression of the complement regulator CD55 is reduced [41]. Genetic studies have also linked polymorphisms in the complement receptor 1 (CR1) gene to susceptibility to the disease, although further population studies are needed to confirm these findings [42]. Peripheral monocytes from patients with sarcoidosis show increased complement receptor expression and elevated phagocytic activity, which may contribute to persistent antigen stimulation and maintenance of granuloma formation [44]. Furthermore, it has been shown that the concentration of activated C5a in the alveolar compartment of patients with sarcoidosis is significantly higher than in other interstitial lung diseases [45]. Based on these observations, it appears that complement system activation products may serve as potential diagnostic and prognostic biomarkers in sarcoidosis.

Pentraxin 3 (PTX3), an important pattern recognition molecule of innate immunity, can activate the classical pathway of the complement system and is thought to play a key role in the immunopathogenesis of sarcoidosis. A study published in 2022 identified PTX3 as a regulator that modulates complement-mediated macrophage activation, thereby limiting the development and progression of granulomatous inflammation [40].

**Figure 5:**
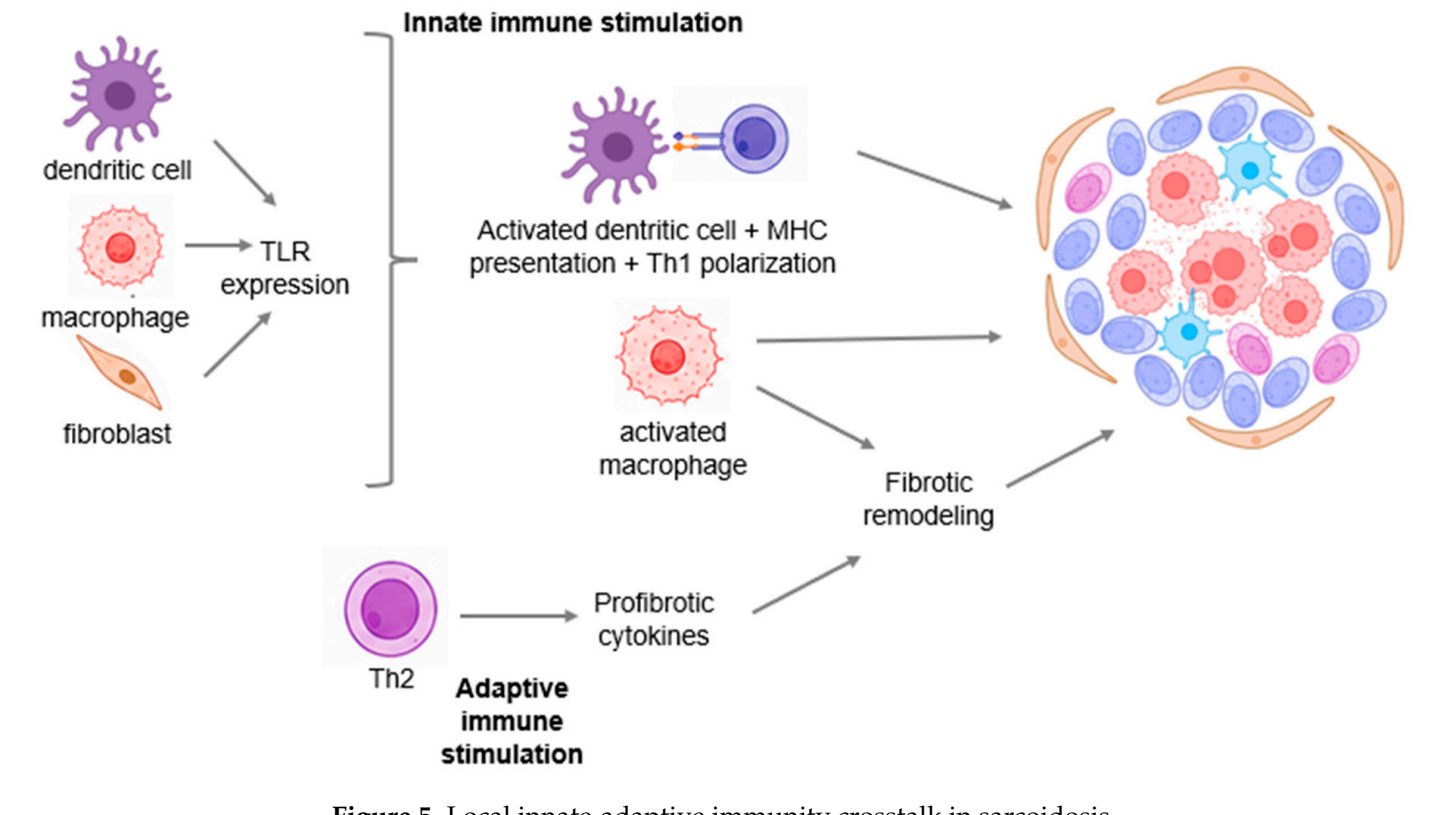
Local innate-adaptive immunity crosstalk in sarcoidosis.

Human genetic data suggest that PTX3 gene polymorphisms influence granuloma size and inflammatory response intensity [40]. These findings point to new therapeutic directions, particularly in the development of PTX3-based biological therapies and complement-inhibiting strategies, which may offer promising alternatives to currently limited treatment options (Figure 5).

Innate immune activation via Toll-like receptor (TLR) promotes a Th1-dominant response, leading to granuloma formation. Sustained immune activation leads to the formation of non-caseating granulomas in the lungs. Within the granulomatous microenvironment, local complement activation, reflected by increased C1q, C3, C5a, and PTX3 levels together with reduced CD55 expression, may amplify macrophage activation and contribute to the persistence of inflammation.

## 4. The Role of the Complement System in the Pathogenesis of Respiratory Malignancies

The development of malignant tumors is the result of a complex, multifactorial and mutation-driven process in which cell cycle regulation is disrupted, leading to uncontrolled cell proliferation. In the case of lung cancer, neoplastic transformation is most often caused by carcinogenic compounds found in tobacco smoke, various environmental toxins, and genetic and epigenetic factors [46]. Lung cancer remains the leading cause of cancer-related deaths worldwide [47].

Tumor cells undergo genetic and epigenetic changes compared to their initial state, and their altered antigen expression makes them recognizable by the immune system. The innate and adaptive branches of the immune system jointly influence the tumor microenvironment, affecting tumor growth, tumor cell selection and immune evasion mechanisms [48].

Previously, the complement system was considered a complementary element in tumor immunology that increases the effectiveness of antibody-based immunotherapies. However, research over the past decade has revealed that unbalanced complement activation is associated with the tumor-associated inflammation and can suppress antitumor immune responses [48].

One study used publicly available databases to compare the concentration of complement components in plasma and lung cancer tissues. The results suggest that complement system imbalance is a pre-neoplastic condition and may have diagnostic and therapeutic relevance [49].

Activation of the cascade can act as a positive or negative regulator in the anti-tumor immune response [5]. Corrales et al. provided the first evidence of the tumor-promoting effect of the system using animal models. In their study, they observed slower tumor growth in cancerous mice using a C5a antagonist. In addition, they found that although C5a did not affect cancer cell division in vitro, it promoted endothelial cell migration and angiogenesis and contributed to the creation of an immunosuppressive environment necessary for tumor growth [50].

Several studies have suggested the use of complement proteins or activation fragments as biomarkers for the diagnosis and prognosis of NSCLC. Previously, C3 levels were identified as a valuable prognostic marker [47]. Meanwhile, the results of another study suggest that the presence of a specific polymorphism (rs2564978) in CD55 (DAF) may contribute to an increased risk of NSCLC and, within this, significantly increase the risk of adenocarcinoma in the Chinese population [51].

Not only the presence of complement proteins, but also the epitopes on their surface may have biomarker value. Proteins associated with lung cancer, such as C9, C4b-binding protein (C4BP), α2-HS glycoprotein and H factor, show epitopes associated with lung cancer [52]. Tornyi et al. identified three different C9 epitopes (BSI0449, BSI0581, BSI0639) that showed negative, neutral and positive associations with non-small cell lung cancer. Based on these results, C9 proteoforms may also be valuable as biomarkers [46].

Despite significant results, it remains an open question whether complement biomarkers have real clinical value and to what extent they can reliably distinguish between benign and malignant tumors and predict the course of the disease [46].

Overexpression of the C5a receptor (C5aR) has been demonstrated in several tumor types and has been associated with aggressive tumor characteristics and poor prognosis. In addition, increased C5aR levels have been observed in NSCLC patients who experienced disease progression after initial anti-PD-(L)1 therapy (aPD1). It has been shown that the combined use of anti-PD1 and anti-C5aR antibodies has an enhanced anti-tumor and antimetastatic effect in mice with lung cancer. The combination effectively slowed tumor growth and significantly increased survival in lung cancer mouse models, which was associated with the activation of CD8+ T cells [53].

Studies focus on the relationship between NSCLC and the complement system; currently, the role of the cascade in small cell lung cancer (SCLC) is less well known and less researched.

## 5. The Role of the Complement System, T2-Type Immune Response, and Eosinophil Cell Interaction in Respiratory Inflammation

According to a study by Thomas and Lajoie, the complement system, particularly the C3 and C5 components, plays a key role in the development of allergic, T2-type inflammation in the skin, intestines and airways, i.e., in diseases directly related to barrier surfaces in direct contact with the environment [2].

C3a and C5a anaphylatoxins activate antigen-presenting cells, mainly targeting dendritic cells, and promote the T2 polarization of naive T cells, thereby enhancing the production of IL-4, IL-5 and IL-13. These processes sustain inflammatory responses, mucosal hyperreactivity and eosinophil recruitment. In addition, C3a and C5a can inhibit the function of regulatory T cells, promoting T2 dominance [2].

The anti-inflammatory role of IgG4 antibodies in the type 2 immune response is a good example of how complement system activation can influence the outcome of inflammation. While IgE binds to allergens and triggers powerful effector cell activation and inflammatory processes, IgG4 is unable to effectively activate the complement system due to its specific structural properties. This is because IgG4 binds weakly to C1q and Fcγ receptors, thus failing to initiate the classical complement cascade. All this indirectly suggests that the complement system plays a decisive role in the regulation of the type 2 immune response [10].

Ogulur et al. describe in detail how, during the T2 immune response, alarmins produced by epithelial cells (TSLP, IL-25, IL-33) activate T2 cells and ILC2 cells, which trigger eosinophilic inflammation by producing IL-4, IL-5 and IL-13. IL-5 plays a key role in the maturation and tissue accumulation of eosinophils, while IL-4 and IL-13 contribute to B-cell IgE production and damage to epithelial barriers. In respiratory diseases (such as asthma or chronic rhinosinusitis), eosinophil infiltration, mucus hypersecretion and airway remodeling are the main consequences of T2-mediated inflammation [11].

**Figure 6:**
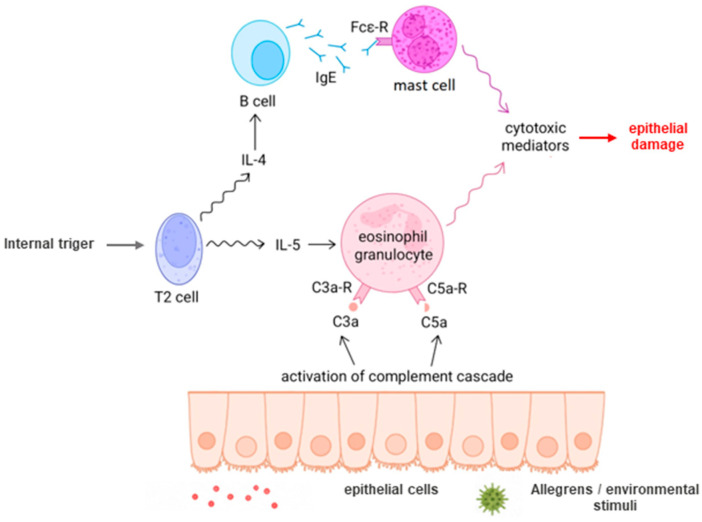
The role of complement-derived anaphylatoxins in the amplification of type 2 immune responses. Complement activation amplifies type 2 (T2) immune responses in the airways. Complement-derived C3a and C5a act on eosinophils via C3aR and C5aR, further enhancing their activation. Cytotoxic mediators released from eosinophils and mast cells induce epithelial damage, which in turn promotes additional complement activation. Together, complement activation and T2 cytokine signaling form a positive feedback loop that sustains chronic airway inflammation.

Complement system activation and T2-mediated inflammation reinforce each other. C3a and C5a promote IL-5 production and thus eosinophil recruitment to lung tissue, while mediators produced by eosinophils (e.g., cationic proteins, TGF-β) enhance tissue fibrosis and epithelial damage, which induces further complement activation. Anaphylatoxins and cytokines involved in T2 immunity together form a positive feedback loop that maintains chronic inflammation in the airways (Figure 6).

## 6. Discussion and Areas of Further Research

In recent years, research into the complement system has expanded our understanding of respiratory disease immunopathology. The traditional view that the complement system functions merely as a defense mechanism against pathogens is now outdated; it is recognized that well-regulated system is essential for maintaining homeostasis, while its dysregulation leads to diverse pathological processes.

In infectious diseases, particularly influenza and COVID-19, excessive complement activation drives overproduction of inflammatory mediators and anaphylatoxins, contributing to acute lung injury and microthrombotic complications. However, the precise contribution of individual complement pathways and dynamics of their activation remain incompletely understood, and the translation of current knowledge into clinical practice also remains challenging [54]. Complement-targeted therapies, such as eculizumab or ravulizumab, have shown promising results in early clinical observations, although their clinical application remains limited and requires further validation.

In chronic diseases including as asthma, COPD and sarcoidosis, distinct patterns of complement activation contribute to chronic inflammation, tissue remodeling, and eosinophil infiltration. Evidence suggests that complement activation is not uniform but rather context and phenotype dependent [9]. The signaling pathways mediated by C3a and C5a are closely linked to the Th2-type immune responses, supporting the concept of a complement-T2-eosinophil axis as a central mechanism in airway inflammation that shapes both neutrophil and eosinophil inflammatory endotypes.

In lung cancer, the complement system exhibits a dual role, exerting both anti-tumor and tumor-promoting effects. C5a–C5aR signaling contributes to the development of an immunosuppressive tumor microenvironment and angiogenesis. Preclinical models combining PD-1 and C5aR blockade suggest a novel direction for systemic immunotherapy, although clinical translation remains to be established. The role of complement in respiratory disease should be interpreted within a broader immunological and temporal context, as its effects are highly dependent on disease stage, tissue environment, and cellular activation. Complement activation is generally associated with pro-inflammatory outcomes in acute settings, but evidence suggests additional regulatory and homeostatic functions depending on the inflammatory milieu and receptor expression patterns on immune cells. This functional diversity is further supported by recent insights demonstrating that complement activity is highly compartmentalized, operating not only systematically and locally within tissues but also intercellularly as a part of the compleosome, where it regulates fundamental cellular processes such as metabolism and survival [55]. The complement system could be viewed as a multilayered network rather than a linear inflammatory cascade.

Changes in circulating and locally produced levels of complement components, as well as their epitope profiles, offer promising opportunities for biomarker development and patient stratification. Furthermore, assessment of complement activation directly at the airway surfaces may provide more accurate insights into local disease activity than systemic measurements alone. Non-invasive methods, such as measurements in exhaled breath, have great importance as they are well standardized, easy to use, repeatable, and have no (or minimal) adverse effects [56,57]. Kokelj S et al. demonstrated activation of the complement and coagulation systems in the airways of asthmatic patients compared to healthy subjects by detecting complement factors and other related molecules in exhaled breath particles [58]. This study provides evidence for the potential of exhaled breath biomarker measurement to assess complement activation in the respiratory tract lining fluid. Further studies are required to demonstrate the feasibility of such monitoring to prove that detecting complement activation locally from the airway surfaces could be added to other exhaled breath biomarkers that are already in use for diagnosing and monitoring respiratory diseases [59].

Overall, the complement system represents a central, context-sensitive yet underrecognized regulatory hub in the pathophysiology of respiratory diseases rather than a uniform unidirectional effector pathway. Its role varies across disease states, inflammatory endotypes, and tissue compartments. Future research should focus on clarifying underlying mechanisms, clinical validation and optimizing the safe use of complement-mediated therapies. The diagnostic, prognostic and therapeutic use of complement components may significantly advance the immunomodulatory approach to respiratory diseases.

## Data Availability

No new data were created or analyzed in this study.

## Abbreviations

The following abbreviations are used in this manuscript:

AHR	airway hyperresponsiveness
aHUS	atypical haemolytic uraemic syndrome
ALI	acute lung injury
aPD1	anti-PD-(L)1
ARDS	acute respiratory distress syndrome
BAL	bronchoalveolar lavage
C1	complement factor 1
C2	complement factor 2
C3	complement factor 3
C3a	complement factor 3 fragment a
C3b	complement factor 3 fragment b
C4	complement factor 4
C4BP	C4b-binding protein
C5	complement factor 5
C5a	complement factor 5 fragment a
C5aR	C5a receptor
CD35	cluster of differentiation 35
CD55	cluster of differentiation 55 (also known as decay-accelerating factor /DAF/)
CEP	chronic eosinophilic pneumonia
COVID-19	coronavirus disease 2019
COPD	chronic obstructive pulmonary disease
CR1	complement receptor type 1 (CR1) also known as C3b/C4b receptor or
CT	computer tomography
DAF	Decay-Accelerating Factor (also known as CD55)
FeNO	Fractional exhaled Nitric Oxide
HA	haemagglutinin
IL-25	interleukin-25
IL-33	interleukin-33
MAC	membrane attack complex
MBL	mannose-binding lectin
NA	neuraminidase
PTX 3	Pentraxin 3
SARS-CoV-2	Severe Acute Respiratory Syndrome Coronavirus 2
SCLC	small cell lung cancer
T2	Type-2
TGF-β	Transforming Growth Factor-beta
TMA	thrombotic microangiopathy
TSLP	Thymic Stromal Lymphopoietin
